# The Search for Significance: A Few Peculiarities in the Distribution of *P* Values in Experimental Psychology Literature

**DOI:** 10.1371/journal.pone.0127872

**Published:** 2015-06-10

**Authors:** Michał Krawczyk

**Affiliations:** University of Warsaw, Dluga 44/50, 00–241, Warsaw, Poland; Stanford University, UNITED STATES

## Abstract

In this project I investigate the use and possible misuse of *p* values in papers published in five (high-ranked) journals in experimental psychology. I use a data set of over 135’000 *p* values from more than five thousand papers. I inspect (1) the way in which the *p* values are reported and (2) their distribution. The main findings are following: first, it appears that some authors choose the mode of reporting their results in an arbitrary way. Moreover, they often end up doing it in such a way that makes their findings seem more statistically significant than they really are (which is well known to improve the chances for publication). Specifically, they frequently report *p* values “just above” significance thresholds directly, whereas other values are reported by means of inequalities (e.g. “*p*<.1”), they round the *p* values down more eagerly than up and appear to choose between the significance thresholds and between one- and two-sided tests only after seeing the data. Further, about 9.2% of reported *p* values are inconsistent with their underlying statistics (e.g. *F* or *t*) and it appears that there are “too many” “just significant” values. One interpretation of this is that researchers tend to choose the model or include/discard observations to bring the *p* value to the right side of the threshold.

## Introduction

Statistics is the corner stone of nearly all empirical science. It provides the essential tools to distinguish between the systematic and the incidental. However, the power of statistics brings with it the possibility of abuse. Most authors are well aware of the fact that the statistics in their papers (notably: the statistical significance of the effects they are reporting) may substantially affect their publishability (“publication bias”) and also their impact. If so, we can expect them to employ what we can call strategic reporting: to include those results and in such a way that favors their theories [1,2].

For the purpose of this study a large data set of statistics (*p* values and otherwise) reported in about 5’000 papers has been collected and investigated. I find evidence suggesting that various types of decisions (e.g., whether or not to round given number and whether to use a one- or a two-sided test) are made strategically, that is, with the aim of presenting the results as “more significant”. I also find that in about 9.2% of the cases the reported *p* value is inconsistent with the underlying statistic (e.g., the *F* statistic). Finally, there seems to be “too many” “just significant” *p* values. These patterns seem to result from what Simmons et al. would refer to as using *researcher’s degrees of freedom* to the full, with the goal of placing the value below the threshold. It needs to be emphasized that my data do not provide evidence of any conscious, fraudulent behavior (let alone suggest which authors may have committed it). It is more likely that some researchers may fall prey to self-serving bias, concluding that his or her choices that happened to give *p* = .048 (rather than *p* = .065) are actually the right ones.

The remainder of this paper is structured as follows. In section 2, I analyze the publication bias and the perverse incentive it creates for the researchers. I also try to answer the question whether they are likely to give in and if so, whether there is indeed enough room for strategic choices in data analysis. In section 3 I describe the design of my empirical study, the data set collected, predictions and results. Section 4 contains a brief discussion and some conclusions.

### Publication Bias and Its Incentive Effects

One of the ways in which statistics turns out to generate rather undesirable effects has been called the publication bias and documented in dozens of studies [3, 4, 5]. We speak of publication bias when statistical significance of an effect reported in a paper (in other words: whether or not the null hypothesis can be rejected) strongly affects the chances for publication: "negative" results are much more difficult to sell, controlling for other qualities of the study. Of course, failure to reject the null may result from deficiencies of the design or data set. For instance, if the theory from which the alternative hypothesis has been derived is highly implausible in the first place, then the "null result" is not very interesting. Similarly, it may be due to researcher's inability to collect enough data or the data being too noisy (particularly when proxies have to be used instead of the variables of interest). To the extent that it is difficult to establish the exact reasons of the null result in every single case, it is a reasonable strategy to be prejudiced against them. Further, the alternative hypothesis is often more "attractive", such as e.g. in the case of research on functional differentiation of the brain hemispheres.

However, the publication bias has long been recognized as highly undesirable, as it leads to a blurred picture of the strength of an effect (although many techniques have been developed to correct for the publication bias when aggregating past results, particularly by means of meta-analyzes, Stanley (2005)). In an extreme case, if out of 20 studies investigating some non-existing difference, only, say, one that happens to deliver significance at 5% level will be published and read, we obtain a completely distorted view. This may, for example, lead to costly policies being based on unwarranted findings of effectiveness of some measures. Further, if negative results are not made public, other researchers may waste time and money challenging the same, correct, null hypotheses over and over again. As Ferguson and Heene [6] recently pointed out, the problem also severely affects effectiveness of replications as a tool of validation of scientific findings—they are but meaningless if only successful attempts are reported.

The negative consequence of publication bias is said to be reinforced by the so-called file-drawer problem [7]. Indeed, if researchers realize that their negative results have poor chances to be published, they have little incentive to ever describe them properly and submit to a journal.

I argue, however, that the incentive effects of the publication bias may be more perverse and more severe. With increasing pressure to publish, the researchers may start considering bending the rules to assure statistical significance of the findings [8]. The two questions we need to ask are, first, whether they would be willing to do so and, second, whether it is feasible. These are addressed in the next subsections.

### 2.1 QRP—survey evidence

Reliability is a crucial if not defining feature of all research activities. We therefore should expect researchers to be extremely careful in the way they design, conduct and report their studies. However, we have some evidence that this is not always so [9,10].

Survey evidence on how researchers use their degrees of freedom in data analysis and reporting is somewhat limited. To get some additional insight, we can take the frequency of other types of (self-reported) substantial violations of the proper standards of scientific conduct as a lower bound.

In a large-scale study of scientists funded by the National Institutes of Health [11] it was found that 0.2–0.3% admit to falsifying research data. Some 11–12% admit "withholding details of methodology or results" and about 15% confess "dropping observations (…) based on a gut feeling (…)"

In the field of psychology the figures are often even more frightening [12]. More than 20% admit to “‘rounding off’ a *p* value (e.g., reporting that a *p* value of .054 is less than .05)”. About 60% admit to having used what we may call endogenous study stopping rules (i.e. collecting the data until the effect is significant, obviously without reporting that such a rule had been adopted [13]. Finally, some 40% have sometimes decided “whether to exclude data after looking at the impact of doing so on the results”. The prevalence rates that subjects anticipated among peers were even higher.

### 2.2 Questionable data analysis and reporting

Questionable practice in data analysis may at first sight seem a risky venture, as potential consequences are severe. However, the odds of actually having to suffer them are low.

Suppose now that you run a study testing some H0 against some H1 and, regrettably, the *p* value of what you considered the most informative test turns out to be, say, .05351. (thus above the standard significance threshold of 5%). What options are left open if you want to sell it as a significant result?

Assuming you want to stay away from outright fraudulent practice (such as data falsification and misreporting of the relevant statistic or just the *p* value associated with it), one attractive possibility is HARKing (Hypothesizing After the Results are Known) [14]. If originally the effect was expected to be there for all subjects (but it turns out not to be the case), after reconsideration you may come to a conclusion the really appropriate test of the theory is whether it shows for, say, females, risk-averters or individuals who score highly on the neuroticism scale (depending on the kind of data you have and in which group the effect turned out to be greatest). Clearly, such practice easily leads to highlighting randomly occurring, unstable effects.

Sophisticated data analysis provides a myriad of ways: run a tobit or a probit, include or exclude outliers, include more or less control variables etc, always choosing the option that yields the most significant results. In Ronald Coase's words, “if you torture the data long enough, nature will confess”. This problem has long been recognized, of course, and readers have learnt to take the authors' assertions with a pinch of salt. As [15] puts it, "(…) in fact, all the concepts of traditional theory, utterly lose their meaning by the time an applied researcher pulls from the bramble of computer output the one thorn of a model he likes best, the one he chooses to portray as a rose. The consuming public is hardly fooled by this chicanery".

When field data is used, it is often necessary to use proxies for the variable of interest. Sometimes there is simply more than one dependent variable in the data. The author may then report the one that "works best" where two or more options were initially considered.

The possibilities seem to be even richer for experimentalists. There is anecdotal evidence of researchers reporting only some of the experimental sessions, all of which, it would seem, provide relevant data. More than one in four psychologists surveyed in [12] admit to “failing to report all of a study's conditions.” More fundamentally, especially in experimental economics, it is often not well-defined what constitutes an "experiment" [16]. Similarly, it seems that many (if not most) researchers decide whether or not to run additional sessions after seeing the data. While it may be a reasonable strategy (e.g. it seems to make little sense to continue spending money if early sessions already provide very strong results), the researchers should carefully spell it out and calculate what the effect for the reported *p* values is.

Many of the more subtle practices mentioned above will typically result in *p* value being just below the significance threshold. Such "minimal" changes are probably more tempting, less ethically questionable, easier to perform and more difficult to detect. It appears therefore promising to focus on the "just significant" results, the main hypothesis being that we will observe “too many” of them.

Even if the case the author is not willing to employ any of these options, there are still choices to be made about the mode in which the value is reported. First, she chooses whether to give the value to the reader directly (“*p* = …”) or by means of an inequality (comparison with some conventional significance threshold, “*p<*.*05*”). I will call it “the choice of sign”.

Next, if the author had decided to report directly, she has to choose how many decimals to include. For example she can report her *p* value as equal to .05351, .0535, .054 or simply .05. I shall refer to this as “the choice of precision”. It is clear in the example above that reporting just two digits is most appealing, and reporting four seems better than three, as the latter involves the greatest, thus “least-significant” number.

If the author chooses to report the value by means of comparison (inequality), some specific threshold needs to be selected and the sign reported accordingly. In our example, she can for example report “*p*<.1” or “*p*>.05”. This will be called “the choice of threshold.” Here, the first option appears most attractive.

Related to this, for some tests (e.g. *t*-tests), it is possible to choose between a one-sided (one-tailed) and a two-sided test. This is another way of choosing a suitable threshold, as significance in one-sided test at 5% corresponds to significance at 10% in a two-sided test. Gerber et al. [17] report some evidence that authors may indeed be making such choices strategically.

To sum up, the ways to manipulate the data in order to achieve statistical significance are abundant and many of them appear to be relatively safe and quite benign. Again, it may well be that after the fact the researcher is truly convinced that the choice that serves his interest best is also optimal from methodological viewpoint.

### 2.3 Previous evidence on distribution of *p* values

While but a few years ago very little was known about empirical distribution of *p* values, the topic has drawn considerable attention recently. An early analysis is [18] that tried to fit different parametric models to the observed distribution of over 3000 (directly reported) *p* values in three top science journals. The authors find their models unable to account for the overly large number of “just significant” findings. [19] comes to the same conclusion investigating meta-analyses of clinical trials.

Gerber and Malhotra [20] analyze the distribution of z-statistics in two leading political science journals. They find an extreme publication bias using a “caliper test”, i.e. comparing the number of observations just below and just above the significance threshold. Their study was based on 137 papers in which hypotheses were manually matched with statistical tests. Similar findings have been reported for top journal in sociology [21] and economics [22]Most recent studies seem to show that this tendency may have strengthened over the last years [23]

Another closely related finding comes from [24]. These authors focus on one aspect of the analysis presented here, namely inconsistencies between *p* values and their underlying test statistics. Based on 281 papers in six journals they conclude that some 18% of statistical results contain an error, this figure being higher in low-impact outlets.

Very recently a closely related study was independently run [25], in which “too many” just-significant *p* values in three leading psychology journals (including the Journal of Experimental Psychology: General that I also investigate) are reported. What distinguishes the approach taken here from theirs is that I analyze more journals in a longer period, thereby have 50 times more observations and cover all *p* values, not just those in the 0.01-.1 range; perhaps most importantly, these authors only analyze the joint distribution of *p* values, no matter whether they are directly reported or re-calculable from reported F or t statistics, whereas my paper emphasizes seemingly strategic ways in which statistical results are reported, including the choice or precision and threshold, as explained before.

## Materials and Methods

The data set consists of over 135’000 records. The data have been harvested by means of computer-based search from all volumes of the five Journals of Experimental Psychology in the period January 1996-March 2008. These are: Journal of Experimental Psychology: Animal Behavior Processes, Journal of Experimental Psychology: Human Perception and Performance, Journal of Experimental Psychology: General, Journal of Experimental Psychology: Applied and Journal of Experimental Psychology: Learning, Memory, & Cognition. This choice of the data set requires some justification.

I need to stress that I do not think that psychological science is somehow particularly prone to arbitrary or self-serving choices in data analysis and reporting. There is certainly no reason to purport that psychologists are less reliable than other researchers or more susceptible to self-serving bias. However, as suggested in previous subsections and in [1] it appears that experimental psychology provides substantial room for uncontrollable arbitrary choices in analysis and reporting of data. Further, such practices may generally be safer in behavioral sciences, where unsuccessful replication is less indicative of the quality of the original study (e.g. may always result from the subject pool specificity). Note also that self-admission rates and rating of defensibility of different QRPs were highest among psychologists involved in laboratory/experimental research [12]. Data reported [26] in also show that tendency to publish “positive” results only is greatest in the fields low in the “Hierarchy of Sciences”, including psychology.

There is also an advantage over the closely-related field of economics. There, the publications are full of multivariate regressions, whereas psychologists tend to focus on (non-parametric) tests of treatment effects. We may expect the strategic choices concerning data analysis to be applied to "central" rather than control variables. If we thus wanted to use the data from economics papers, we would either have to distinguish between these two types of variables in thousands of papers or end up with a lot of noise. Besides, the common habit (actually: requirement put forward by the publishers) in psychology to actually report the *p* values makes the data collection much easier. All in all, experimental psychology may give the speculated (subtle) effects the best chance to show up.

Regarding the ranking of the journals, it is possible that there is less (detectable) arbitrary and self-serving choices in the data analysis process in studies submitted to top outlets. Further, referees and editors are likely to be doing a better job. On the other hand, it may take more to make it to a really good journal, whereas the “raw”, negative findings would be good enough for a mediocre outlet. While it may be difficult to compare the prevalence, there is clearly a difference in the implications: it is important to establish whether we can at least fully trust the statistics reported in top journals.

The data set consists of the following variables: title of the journal, volume, identification number of the paper, a categorical variable showing which sign is used (" = ", ">" or "<") and, of course, the *p* value (with precision varying from .1 to .00001) itself. Further, for some 27’000 entries I also have the underlying statistic (*F*, *t* or *χ* ^2^) (other types, less frequent than these three are not analyzed) i.e. its name, degrees of freedom and value—these are only available if they directly precede or follow the *p* value (“(*F*(*x*,*y*) *= z*, *p = w*”). For these cases it is possible to re-calculate the *p* value and compare it to the one being reported. More precisely, because the underlying statistics are rounded, I can only calculate the upper and lower end of the range in which the true *p* value lies. Typically, however, this gives a *p* value up to three or more decimals, i.e. more precisely than the authors report themselves (even if the *p* value is reported directly and not by means of “*p*<…”).

Entries in 85 randomly selected papers have been verified manually. I have oversampled entries in which the *p* value was inconsistent with the value of the underlying statistic. While bits of information (such as the number of observations) were missing in isolated cases, only one type of error has been found more than once in this sample, in which very high values containing a comma were incorrectly reported (e.g. the software recorded 1 instead of 1,324.2). All values of the *F*, *t* and χ^2^ that had no decimal part and were inconsistent with the p value have thus been manually verified. It has also been confirmed that, with entirely incidental and unsystematic omissions, *p* values and *p* values only (along with underlying statistics) are being collected.

It should be stressed that I do not have any further information on the type of study, sample being used, the content of the hypotheses etc. (e.g. it could even be that some of the entries refer to quoted findings from other papers, though no such case has been found in the process of manual verification). It is also clear that many statistical results have been overlooked, e.g. those reported in words. This is in stark contrast with earlier studies on issues related to publication bias, where a much smaller sample of publications related to one selected issue would be much more carefully investigated.

## Results

### 4.1 Overview—distribution of *p* values

#### Reported values

We start with presenting the distribution of reported *p* values. We note that the *p* values are rarely reported directly (e.g. “*p* = .391”), even though this is the recommendation of the APA, publisher of the journals in our sample. In fact, only about 16.5% of the entries in our sample involve an equality sign. The rest is reported by comparison with a threshold, usually .001, .01 or .05. Interestingly, in 72.8% of the cases, the obtained *p* value is lower than the threshold (“*p*<.05”) and only in 10.5% it is higher, possibly because the distribution of the originally obtained *p* values is highly skewed (with sharply decreasing density, i.e. most values located very close to 0). Indeed, it has been shown [27,28] that generally speaking a downward sloping distribution is to be expected when at least some alternative hypotheses are true. To the best of my knowledge, there is little guidance in theory as to the exact shape of this distribution in such a heterogeneous sample. Clearly, the publication bias that favors reporting low *p* values only may have contributed to the skew.

**Fig 1:**
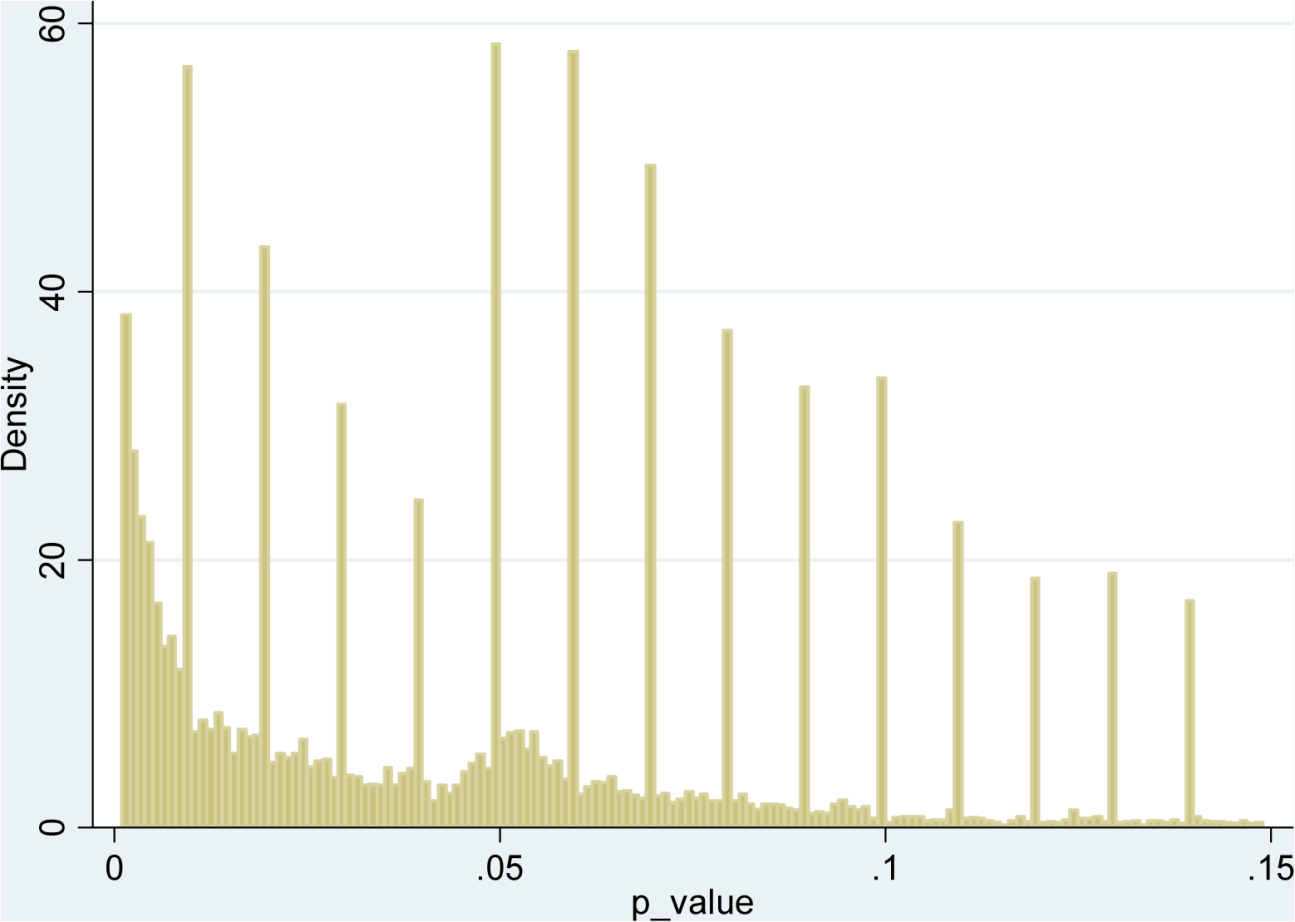
Distribution of directly reported *p* values (restricted to .001–.15).

Fig 1 presents the distribution of directly reported *p* values. More precisely, only values between .001 and .15 are depicted in the figure. Greater values are infrequent and smaller—very frequent. Omitting these values makes the most interesting data patterns more clearly visible. Obviously, the high spikes correspond to the fact that many *p* values are rounded to .01, .02 etc. We see that the density is generally decreasing; however, contrary to what might be expected given the prevalence of “*p*<…”, there is no sign of publication bias in the form of a sharp drop at conventional significance thresholds of .01 or .05. On the contrary, an interesting structure around .05 is clearly visible—there are “extra” values just below and especially just above this conventional threshold.

**Fig 2:**
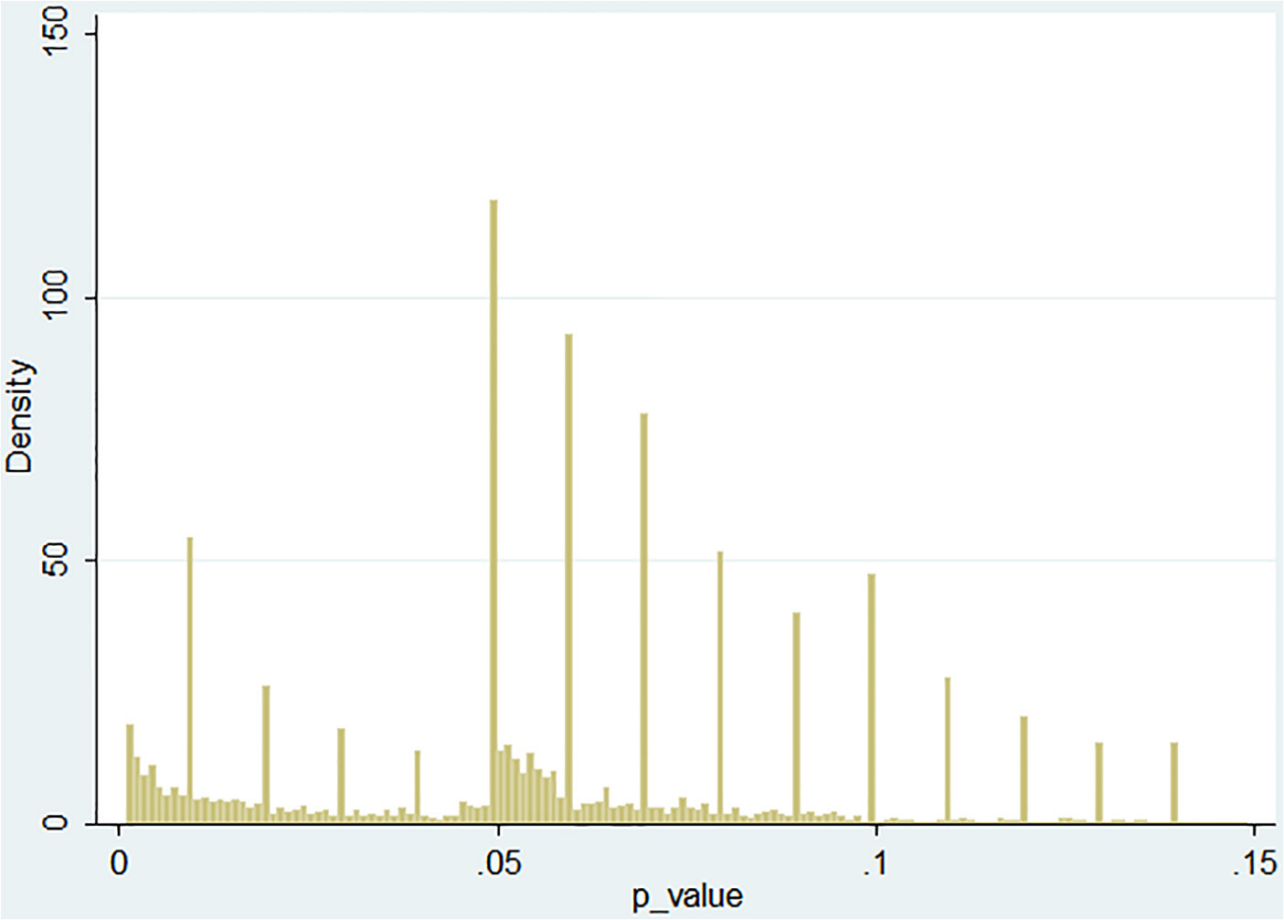
Distribution of directly reported *p* values (restricted to .001–.15, equal weight of each paper).

As a referee pointed out, it is worthwhile to check if these patterns could merely be due to a small subset of papers that contain very many values around the threshold. As Table 1 shows, there are very few such papers. Fig 2 is analogous to Fig 1, except that this times weights are used that are inversely proportional to the total number of *p* values directly reported in given paper. There are still extra values just below the threshold, while the number of extra values just above is actually dramatically stronger.

**Table 1:** Distribution of number of “just significant” (.045–.050] and “almost significant” (.050–.055) directly reported *p* values per paper.

	Almost significant *p* values					
Just sig. *p* values	0	1	2	3	4	Total
0	2,287	217	29	7	1	2,541
1	131	17	4	0	1	153
2	25	6	2	1	0	34
3	7	2	0	0	1	10
4	1	3	0	0	0	4
5	1	0	0	0	0	1
6	1	0	0	0	0	1
Total	2,453	245	35	8	3	2,744

This bi-modality is confirmed by means of kernel smoothing and “bump-hunting” methodology [29], results of which are available from the author. Multi-modality is always a perplexing feature of the data that calls for an explanation [30]. Often it results from a mixture of two different distributions. In fact, I have mentioned the distinction between the "central" and the "control" variables, which is difficult to make without carefully reading the papers. One crude proxy could be based upon the assumption that “central” results come early in the paper. However, the distribution of *p* values appears to be independent of where in the paper they are located. Besides, there seems to be little reason to expect the distribution of *p* values corresponding to "control" variables to have a mode exactly at the arbitrary significance threshold of .05. Thus the distinction between “central” and “control” results is unlikely to explain the observed pattern.

It could also be that there are clusters of types of studies. For example, if experiments on animals involve a lower number of observations (as, contrary to psychology students, some animal species are quite costly when used as subjects) we could expect that they typically result in higher *p* values (e.g. with a mode close to .05), whereas other experiments have lower *p* values (often below .01). If this was the case, however, we should observe different patterns in different journals (e.g. the Journal of Experimental Psychology: Animal Behavior Processes should show *p* values shifted to the right). In fact, the distributions are quite similar across journals, all of them showing the perplexing bi-modality.

The left part of the structure (below the 5% threshold) is consistent with the hypothesis that researchers employ “minimal” data manipulations, ending up in “just significant” results.

#### Actual values

**Fig 3:**
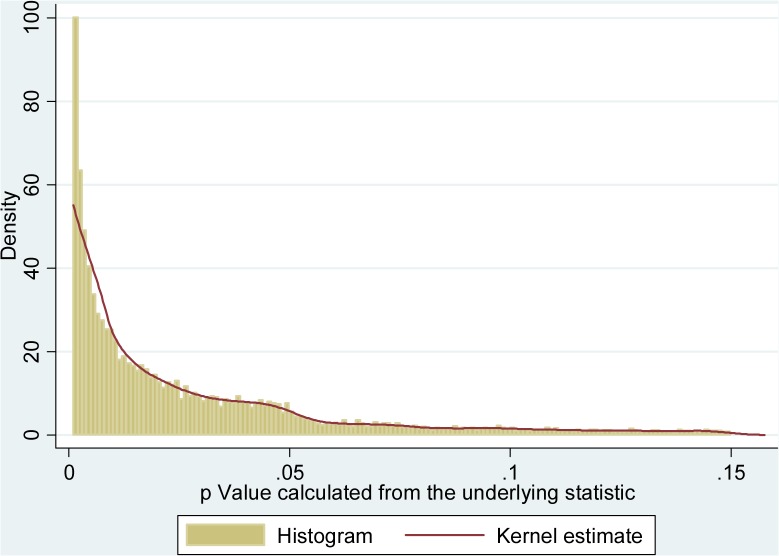
Distribution of re-calculated actual *p* values (restricted to .001–.15).

Another way of obtaining the general picture of the data is to consider the distribution of “true” *p* values re-calculated from the reported underlying statistics (Fig 3, restricted to range .001–.15). It is of interest how this distribution compares to the one depicted in Fig 1, as this difference stems from the (possibly strategic) ways to report the data.

The general decreasing pattern is now more clearly visible, as well as a clear publication bias at the 5% threshold. The artificial spikes have disappeared, as expected, as well as the “bump” around 5%, apparently created by the modes of reporting. Interestingly, however, while density is monotonically decreasing, the decrease is less pronounced just below the 5% threshold. This is confirmed by the overlaid kernel density estimation.

Similar pattern can be observed when we consider different journals or different types of statistics separately. Of course, it is possible that the distribution depicted in the figures emerges naturally. However, the fact that “additional values” appear just below the threshold is also consistent with the idea that authors end up selecting, among the slightly different *p* values coming out of alternative specifications, the ones that are significant.

**Fig 4:**
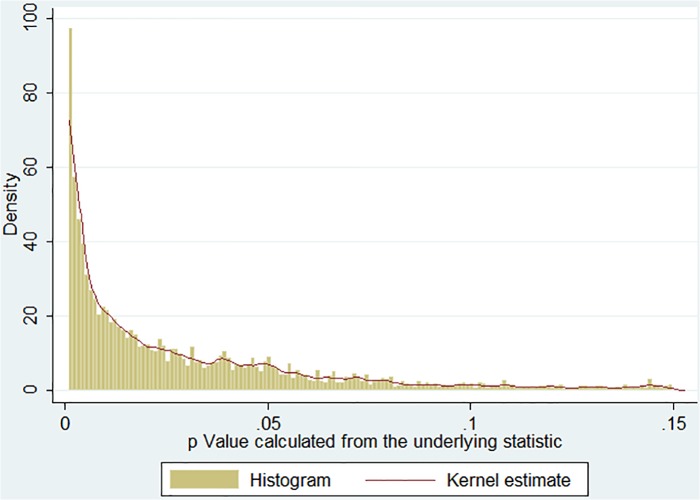
Distribution of re-calculated actual *p* values (restricted to .001–.15, equal weight of each paper).

Again, considering the fact that different *p* values in the same paper cannot be considered independent, one may wish to check if the distribution changes when weights equal to the inverse of the number of calculated *p* values within the paper are used. As can be seen in Fig 4, the pattern remains identical.

In the next subsections I will try to find out, basing on the notions of strategic data reporting, where these data patterns, particularly bi-modality of the distribution of reported values and the differences between reported and calculated *p* values come from.

4.2 The mode of reporting**The choice of sign.** Suppose the author is trying to convince the reader that the finding being reported is significant. As was suggested before, if the *p* value is below some significance threshold, say, 5%, it suffices to report “*p*<.05”. If it is above, then seeing “*p*>.05” the reader thinks it can be anywhere between .05 and 1. Thus if the value is closer to the lower end of this interval, say .0687, it seems a better strategy to report it directly.

**Hypothesis:** *(Strategic choice of sign) (1) Papers will combine the use of equality (with exact* p *value) and inequality (with threshold value) signs*. *(2) "Almost significant" values will be most likely to be reported directly (3) Values below the threshold will be least likely to be reported directly*.

**Result:** (1) Different reporting modes are used within the same paper: nearly half the papers report both some equalities and some "smaller than". Importantly, it is not about very low values (it is natural and actually recommended by the APA to write "*p*<.001" rather than the actual value). Some 43% of papers report both exact values and inequalities involving numbers greater than .01.

**Fig 5:**
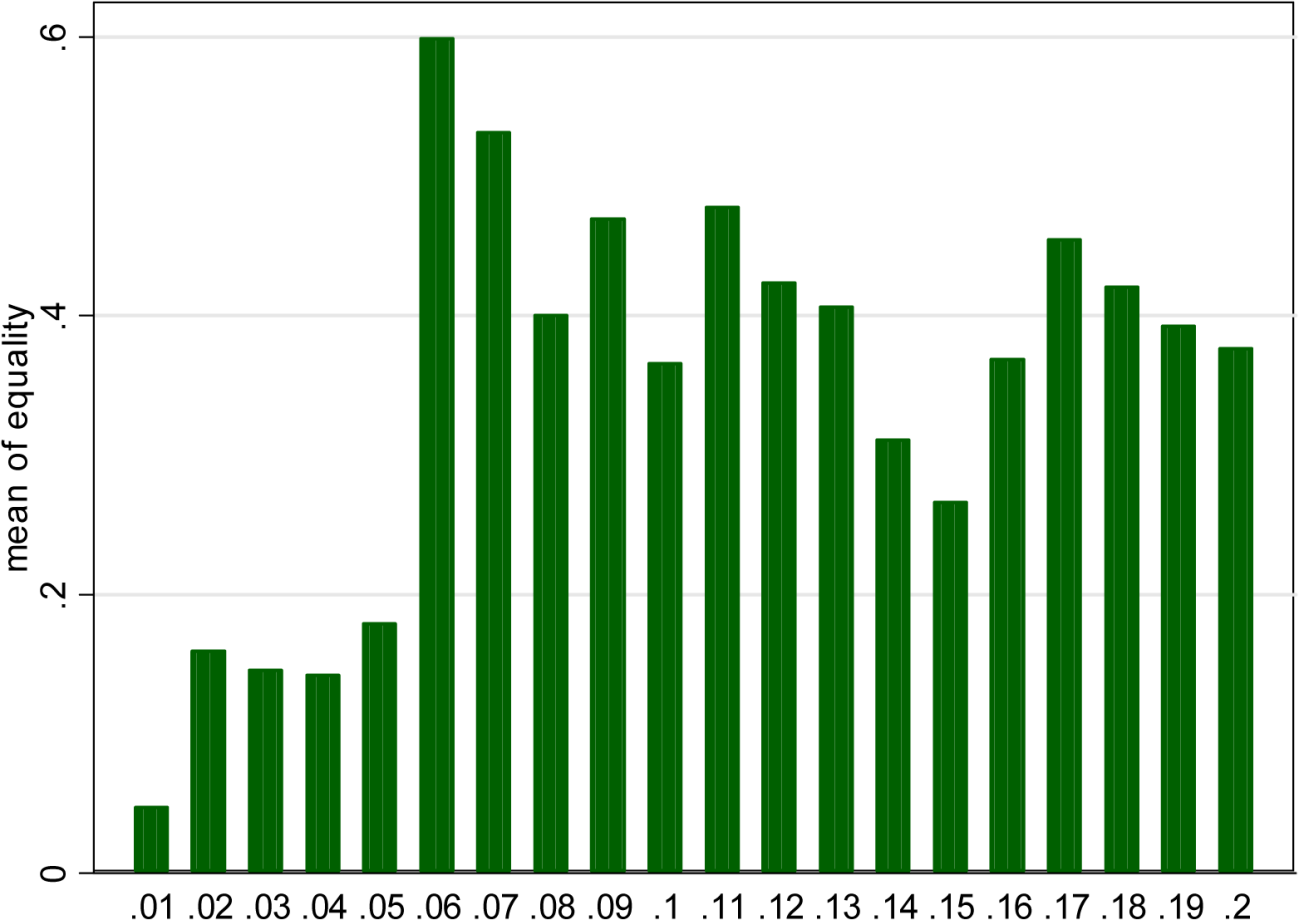
Frequency of reporting directly over intervals of actual *p* value (rounded up).

**Result:** (2) and (3). These points of the hypothesis can be verified by inspection of Fig 5, presenting the frequency of choosing the equality sign, depending on the actual *p* value (rounded up to the nearest percent, only values up to .2 considered for the sake of clarity of the picture).

The hypothesis is fully confirmed. The values that are truly significant (at 5%) are typically reported indirectly. This gives a hint why the “<” sign appears so much more often than “>” in our sample and why the publication bias is not visible in Fig 1.

By contrast, “almost significant” values typically are given directly, with the frequency decreasing slowly as we proceed to higher *p* values. This impression is supported by LOWESS approximation (figure available here: http://coin.wne.uw.edu.pl/mkrawczyk/lowess_for_p_val.pdf).

This finding contributes to the explanation of the right part of the observed structure around the threshold of .05 in Fig 1—the values just above the threshold are simply most likely to be reported directly.

One important point needs to be made here, which will, to varying degree, apply to other findings, namely that it is difficult to distinguish two effects: authors systematically reporting their “almost significant” *p* values directly more often than other values and journals accepting more readily papers in which this happens to be the case (a case for publication bias—selection on reported *p* value). In other words, the prevalence of directly reported “almost significant” values means that either authors have a preference for doing so or that such a practice increases publication chances (or, most likely, both). On a general note, however, I should say that, first, such a selection based on publication bias should not be expected to be very strong, given the fact that there are on average about 30 *p* values per paper in our sample (among those papers that include any statistics at all). The impact of most individual values on the publication probability must therefore be very low. Second, the referees should look at the underlying statistics as well—this would make the selection based on the rounded value still weaker.

#### The choice of threshold

As mentioned before, most values are reported as “*p* smaller than…”. Which of the available thresholds (say: .001, .01, .05, .1) should authors chose? According to the classical hypothesis testing methodology (the Neyman-Pearson paradigm), the critical region of statistic leading to rejection of H0 (thus equivalently: significance threshold) should be determined *ex ante*, i.e. before the data was collected, basing on the strength of hypotheses, number of observations, relative cost of errors of type I vs. type II, etc. If, however, the goal is to persuade the referees, editors and readers that findings are significant, we should expect the author to choose possibly low threshold for which the data admits rejection of the null hypothesis. And this is what we find: for example, among findings that (we know) are significant at .05 and reported by means of inequality, only for .37% the threshold used is higher than .05.

#### The choice of one-sided vs. two-sided tests

Some statistical tests, notably *t*-tests, require the researcher to choose between a one-sided and two-sided critical region. According to standard hypothesis testing methodology, the choice of a one-sided or two-sided test should reflect hypotheses based on theory or previous evidence and thus precede data acquisition. However, as this cannot be verified, researchers may in fact use the one-sided test when it delivers significance where there would be none with a two-sided test and a two-sided test otherwise.

**Hypothesis:** *One sided tests will prevail for true underlying one-sided p values lying in the intervals of* .*0005–*.*001* , .*005–*.*01 and* .*025–*.*05*. *Two-sided tests will prevail elsewhere*.

**Fig 6:**
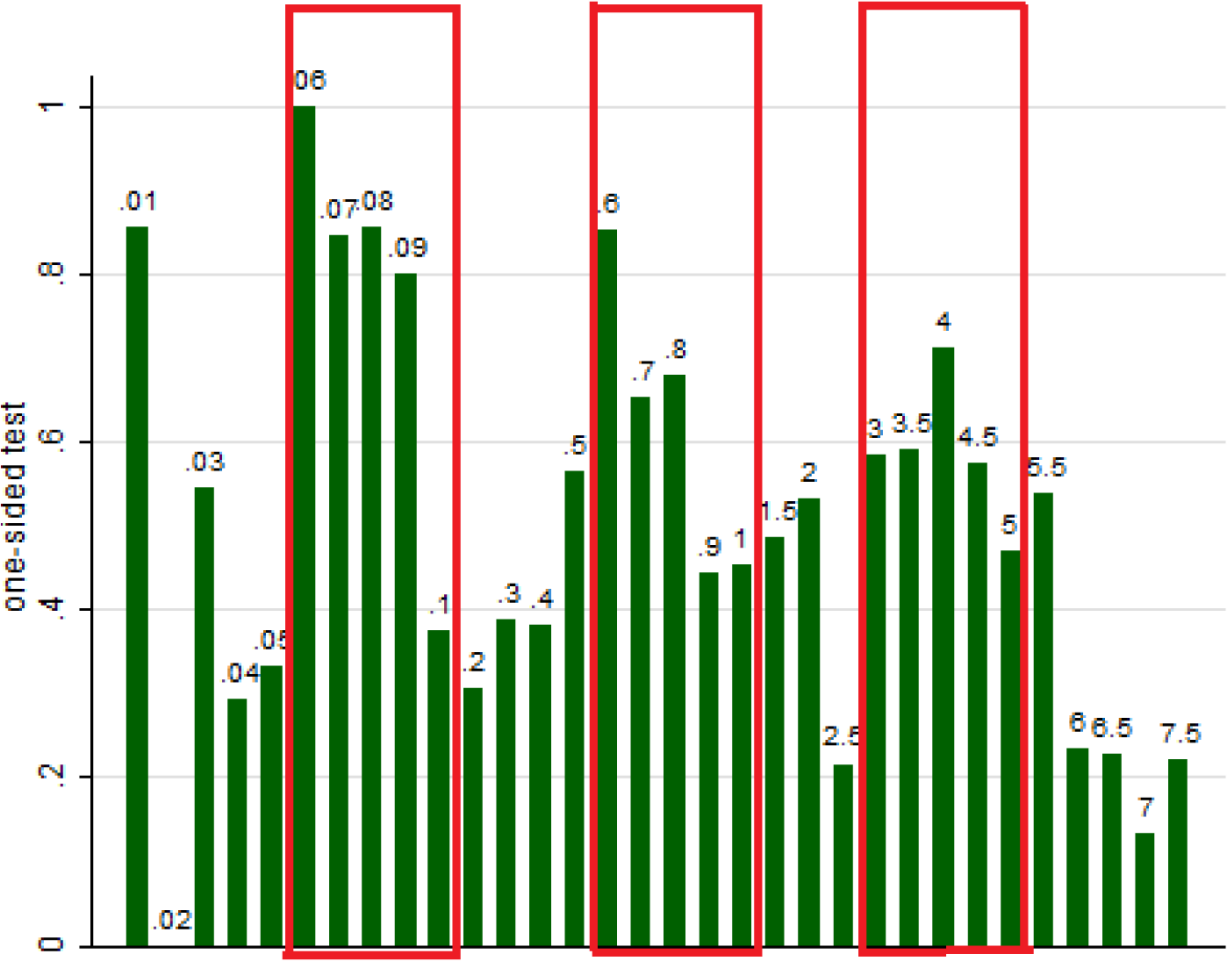
Frequency of use of one-sided tests given actual one-sided *p* values (printed above each bar rather than on the axis and with percent sign omitted for greater readability, rounded up).

Fig 6 shows frequency of use of one-sided tests for different actual *p* values (if one-sided test was to be used), only for the cases when the underlying *t*-statistic is given and it is possible to establish whether one- or two-sided test was used. For values below .001, intervals of length .01% were used, for values between .001 and .01: intervals of length .1%, and between .01 and .075: of length .5%. (The range is restricted for the sake of clarity of the picture, while mixed scale makes the exhibition of the hypothesized effects possible. It also corresponds to the fact that the density of the empirical distribution of *p* values is decreasing—there would not be enough entries to make inference about very short intervals further away from 0.) For example, the fifth bar on the left shows that some 35% of values between .04% and .05% are reported as one-sided. The red boxes contain these bars that we predicted to be high, because they correspond to findings that will be significant at given threshold (.1%, 1%, 5% respectively) only when one-sided test is reported.

**Result:** The data displays all the hypothesized patterns: Indeed, we see that authors tend to use two-sided tests (overall, of all 1376 cases, one-sided test was only used 35,8% of the time), except for the range of .0005–.0009, .005–.0.008 and .025–.045. These intervals overlap almost perfectly with the intervals on which a one-sided test is significant at .1%, 1% and 5% respectively, but a two-sided test is not and thus the use of one-sided test is predicted.

Again, while this is a combined effect of authors’ strategizing and publication bias, the latter is unlikely to account for the large part of it.

#### The choice of precision

Suppose now that the researcher chooses to report the *p* value directly. The data shows that she typically (about 90% of the cases) reports either 2 or 3 decimals. Can we find evidence that the choice which of the two to use is made in a way that makes findings appear more significant?

First of all, it would imply that different entries within one paper have different precision. This, of course would not yet prove that the choice of precision is being made strategically. To investigate that issue, we need to focus on these entries where the underlying statistic is given and thus the actual *p* value can be recalculated. We can only consider those cases, where it can be established whether rounding from three to two decimals raised or lowered the number. For example, if we know that the true *p* value falls in the range of (.0795,.08), it will be, when rounded to two decimals (thus to .08), increased. I call such entries “roundable up”. Similarly if the true value is known to be between .13 and .135, it is “roundable down” (to .13). If, however, the interval of possible correct *p* values is e.g. [.17893,18217] then it is neither roundable up nor down because we do not know whether it is smaller or larger than the rounded value of .18. We can now speculate that values that are “roundable up” will indeed be rounded less often than those “roundable down”.

**Hypothesis:** *(Strategic rounding)*

1. Precision (number of decimals) will vary within papers
2. Roundable up values will be rounded less often than those roundable down,
3. This discrepancy will be particularly strong at the thresholds

Indeed, I find that researchers are not consistent in their choices of precision within one paper. Suppose for example that three decimals are initially obtained (typically from a statistical package). If these are reported with maximum precision, we expect that about 90% of values reported directly in the paper will have three decimals—the remaining 10% will have 0 as the last digit, thus being likely to be abbreviated to just two decimals. About 9% will have two decimals (0 as the last but not second to last digit) and 1% just one decimal.

In fact, only about 61.4% of entries, rather than 90%, have paper-specific maximum precision. Again, this is not about very low values only. If I discard all *p* values lower or equal to .01 and redefine the maximum precision, I find that still just 66% of entries achieve it.

Regarding (2), we can see in Table 2 that the option to round is used 60.6% of the time if it lowers the original value (roundable down) and only 52.4% of the time if it raises it (roundable up) (All the actual *p* values below .01 have been discarded for this calculation, as it may be more natural to round (up to .01) values like .008 than to round (down to 0) values such as .003.;values above. 99 were left out for the same reason). The difference is significant, *p* = .001 (two-sided *t*-test) when individual *p* value is used as unit of observation. As the referees and the editor rightly pointed out, this result must be treated with caution because in principle values within one paper are not independent. The alternative approach would be to treat one paper as one observation and then even that only if it satisfies three conditions: 1) at least one roundable-up, 2) at least one roundable-down, 3) different fractions of actually rounded values for these two categories. The results are similar to those on *p* value level in that 54% of such papers have more rounding in case of roundable-downs than roundable-ups (*p* = .05).

**Table 2:** Actual rounding in roundable downs and roundable ups.

Group	Obs.	Mean	Std. Err.	Std. Dev.
Roundable down	817	.606	.017	.489
Roundable up	694	.524	.019	.500

H0: diff = 0, Ha: diff! = 0; t = 3.192, P > |t| = 0.001.

Further analysis revealed that the effect is partly driven by *p* values around the 5% threshold. Roundable down values just above it, between .050 and .055 are rounded down more than 60% of the time, while those in the neighboring range .055-.060 are rounded (up) less than 40% of the time (*p* = .01, again under the assumption of independent observations).

Interestingly, values just below the intervals, i.e. values in .005-.01 and .0045-.05 have lowest overall probabilities of being actually rounded up. This partly accounts for the left part of the structure around 5% visible in Fig 1. For example, values from the (.40,.45) interval are more often rounded than the values from the (.45,.50) interval.

Again, it is not logically impossible to exclude an alternative explanation of the fact that opportunities to round down seem to be taken more often than opportunities to round up; it could simply result from the publication bias—selection on reported *p* values—because rounding down increases publication chances and rounding up hurts them. However, such an effect would have to be extremely strong to account for the data. To acknowledge this, we may do a little exercise. It will show just how powerful the publication bias would have to be to explain the discrepancy between rounded ups and rounded downs for *p* values in the (.02, .03) range, see Table 3.

**Table 3:** Rounded and not rounded *p* values in the (.02-.03) interval.

	Rounded	Not rounded	Total
Roundable down (.020-.025)	59 (50.4%)	58 (49.6%)	117
Roundable up (.025-.030)	41 (36.9%)	70 (63.1%)	111

Let us first carefully consider where our sample of, say, *p* values actually rounded down to .02 (there are 59 of them) precisely come from. Among the population of all *p* values that come out of statistical analyses in studies submitted to our journals, they have to satisfy four conditions in order to be observed as “rounded down to .02”. That is, they must:

- indeed be in the interval of .020-.25
- be reported using the equality sign rather than by comparison with a threshold
- be actually rounded (down) rather than reported with three or more decimals
- be in an accepted paper, given rounding (I make a conservative simplifying assumption here that only the reported, not the underlying true *p* value affects the probability of publication).

Let us denote the probabilities involved in points c. and d. as *P*(*RD*) and *P*(*acc*|.02) respectively. Similarly, to be included in our sample of roundable downs within the same interval that have *not* been rounded, *p* values must satisfy a. and b. as above and additionally c’.: be *not* rounded and d’.: be in an accepted paper, given *no* rounding. The relevant probabilities will be 1-*P*(*RD*) and *P*(*acc*|.020-.025). Using the numbers given in Table 3 we thus conclude that $\frac{P(RD)}{1-P(RD)}\frac{P(acc|.02)}{P(acc|.020-.025)}=59/58.$ (Note that the probabilities associated with points a. and b. cancel out and thus are irrelevant). The second fraction is a measure of publication bias for this range.

Similarly, for the values in the (.025,.30) interval we infer that $\frac{P(RU)}{1-P(RU)}\frac{P(acc|.03)}{P(acc|.025-.030)}=41/70,$ whereby *P*(*RU*) refers to the probability of rounding up of a *p* value that lies within (.025,.30). If we now supposed that authors are equally eager to round up as down, *P*(*RU*) = *P*(*RD*), it would lead to the conclusion that $\frac{P(acc|.02)}{P(acc|.020-.025)}\frac{P(acc|.025-.030)}{P(acc|.03)}=\frac{59/58}{41/70}\approx 1.74.$ Thus the combined effect of two shifts of a single *p* value by less than half a percentage point (from .03 to somewhere between .025 and .03 and from somewhere between .02 and .025 to .02) would have to increase the probability of publication by 74%. Such an extreme publication bias is highly implausible.

#### Strategic mistakes?

In this subsection I investigate the cases in which the recalculated *p* values are inconsistent with the reported ones. To begin with, these instances are fairly common. The reported *p* value falls outside the permissible range (in which it should be given the value of the underlying statistic) 9.2% of the time.

To make it clear, generally speaking we cannot know which part is erroneous—the p value, the test-statistic or perhaps the number of degrees of freedom (df). An anonymous referee made an interesting and plausible suggestion that authors may be particularly likely to misreport the latter. The referee also pointed out that such possibility may be explored by calculating how many discrepancies could be explained by replacing the (possibly incorrectly) reported df with some other integer. I have run a simulation for all the errors involving *χ* ^2^ statistics and directly reported *p* values, with “alternative” df values running from 1 to 1000 (less than 1% of *χ* ^2^ statistics in my sample have even higher df). It turns out that in 76.8% of these entries reported *p* value would not be correct for any df. It thus appears that only a small minority of errors could have arisen because only the df was misreported. A similar exercise could be done for *F* and *t* statistics, the additional complication being that in the former we would have to allow in simulations for errors in both d1 and d2 and in the latter—for tests being one- or two-sided.

Another explanation of the high number of mistakes, also suggested by the referee, is that authors may inadvertently paste the same *p* value twice (i.e. by mistake report the figure corresponding to another test). To verify how often this may be happening I have created variable “repetition” taking value of 1 if and only if the same *p* value, greater than .01, is reported directly (i.e. using equality sign) more than once in the same paper. It indeed turns out to be a powerful predictor—a whooping 24.9% of such entries are inconsistent. However, because they only constitute 3.7% of all entries, error rate in not-repetitive *p* values remains very high, equal to 8.8%.

**Fig 7:**
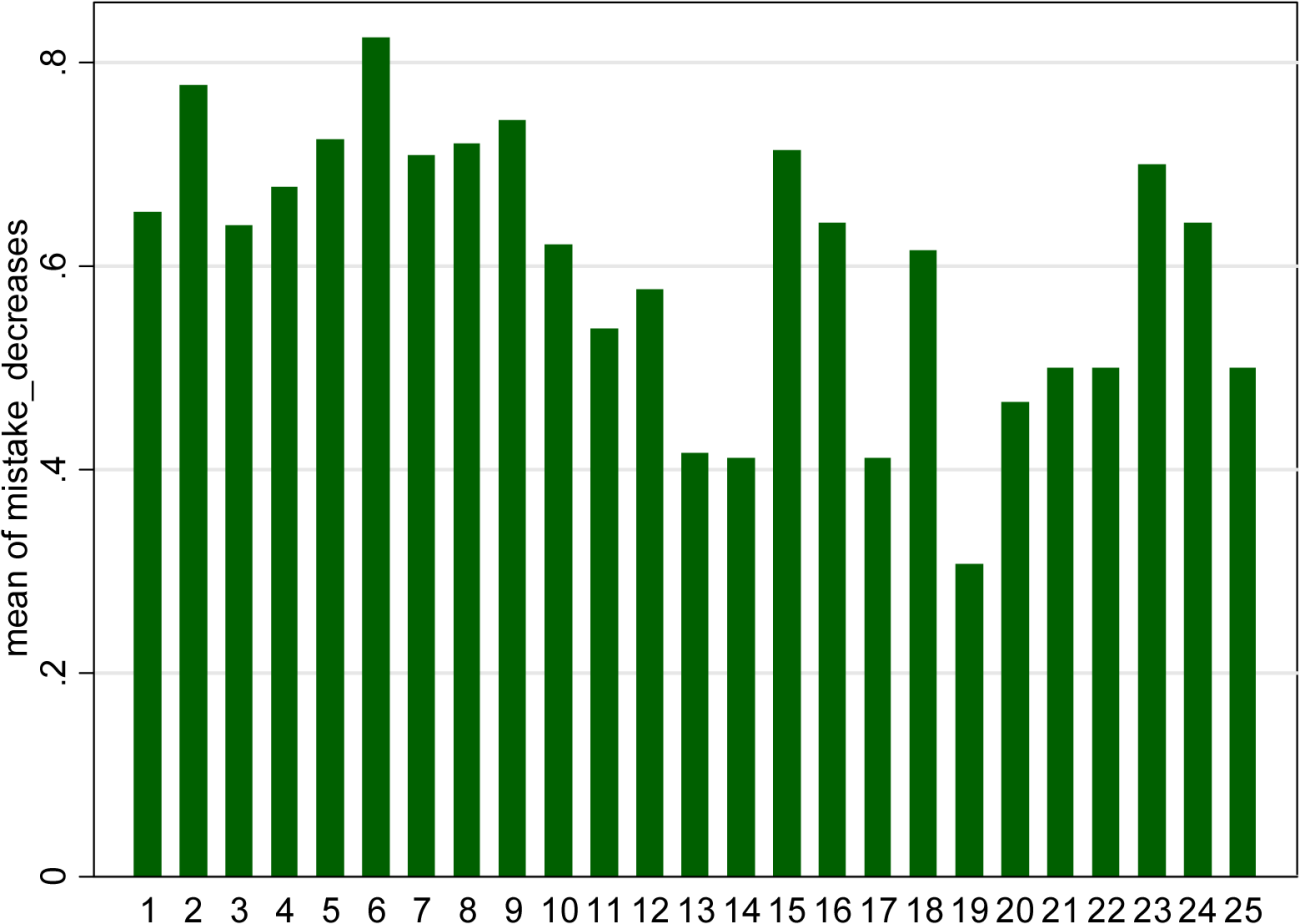
Fraction of mistakes leading to the reported *p* value being lower rather than higher than the actual *p* value, over the calculated *p* value (rounded up, percent sign omitted).

Given that a substantial number of discrepancies seem to be due to misreported *p* values, I further hypothesize that researchers are more eager to double-check when the value to be reported is not to their liking, i.e. implies non-significance. As a result, remaining mistakes would more often than not be self-serving. To verify this hypothesis I investigate how often the reported value is higher than the actual value rather than lower, for each interval of length .01 separately. Inspection of Fig 7 yields interesting observations (note that for clarity of the picture only first 25 intervals are considered.) We note that a mistake typically leads to lower, not higher values: the mean of mistake decreases is substantially above .05 for most intervals, in particular *all* the intervals below the 10% threshold. This is perplexing. As a matter of fact we initially had reasons to expect that most mistakes should lead to reported values being *higher* than true values, at least in the case of low true values. First, there is more “space” for mistakes leading to higher reported value. For example if incorrectly reported values were distributed uniformly, there should be, e.g. just a chance of .054 for the incorrectly reported *p* value to be lower than the original value of .054. Given that most correct *p* values are low, large majority of mistakes would involve over-reporting. Second, the “typical” mistake that I find in the data relatively frequently, i.e. omission of the initial “0” (thus turning .067 into. 67 etc.) necessarily leads to the reported *p* value being higher than the true one.

We also see that largest discrepancy between the two types of mistakes is for the sixth interval, where rounding down is most desirable.

Arguably, most mistakes do not lead to great misrepresentation of the data. For example, the median *p* value among the entries wrongly reported as “*p*<.05” is 0.066. Still, for about 25% of these cases the true *p* value exceeds .1, thus is two times higher than the reported threshold; the mean is equal to .144. Given that mistakes are not extremely frequent and that they sometimes lead to substantial changes in the *p* value, the fact that most of them lead to lower, not higher *p* values, may have well resulted chiefly from selection (publication bias). In other words, I cannot prove that authors selectively overlook mistakes that happen to lower their *p* values, though they would apparently benefit from this tendency. Of course, we do not know either how many of the mistakes are discovered by the referees (and, most probably, lower chances for publication).

As mentioned before, a discrepancy could also arise from the *p* value being correct but the underlying statistic (its value or number of degrees of freedom) being wrong. Under the assumption that it is the *p* value is what attracts most attention, we may expect that such errors are equally likely to go either way. This observation thus only sthrengthens the result.

As pointed out by a referee, it seems instructive to look at the number of mistakes per paper. This distribution is reported in Table 4. It is somewhat reassuring that more than three-quarters of papers are error-free (or almost 90% if we ignore very small mistakes that do not change the second decimal) and papers with very many mistakes are rare On the other hand, note that on average we only have 5.08 entries per paper for which we can re-calculate *p* value from the underlying test-stastic and tell if the reported figure is consistent or not. As a result fractions of mistakes remain high, e.g. one in ten papers has at least one-third of its statistics wrong.

**Table 4:** Distribution of number of mistakes per paper.

Number of mistakes	# of papers	%	Cum. %
0	4101	78.59	78.59
1	613	11.75	90.34
2	236	4.52	94.86
3	113	2.17	97.03
4	53	1.02	98.05
5	34	0.65	98.70
6–10	54	1.03	99.73
11–26	14	.27	100.00
TOTAL	5218	100	

## Discussion and Conclusions

Upon analyzing the *p* values and underlying F, t and χ^2^ reported in top journals in experimental psychology I find some disturbing patterns. One interpretation that correctly organizes these findings is that the authors, being aware of that or not, tend to use their “degrees of freedom” to their advantage, rather than follow any predetermined best practice. In particular, they seem to engage into strategic choice of sign and threshold, strategic choice between one-sided and two-sided tests and strategic rounding. Reported *p* values are surprisingly often inconsistent with the underlying (reported) statistic and these mistakes tend to lower the *p* value.

Without obtaining the original data and re-running all the estimations (which of course could only be done with a much smaller sample) we cannot identify other mistakes or questionable practices. We do however observe an intriguing bi-modality in the distribution of *p* values: next to the mode situated close to 0 we also have a bump centered near the significance threshold of .05. The left part of the bump—“extra” values just below the significance threshold, is not fully explained by any of the identified patters in the way the *p* values are reported. Therefore it persists (in the form of flatter density curve) also in the distribution of the actual *p* values, re-calculated from the underlying statistics. One possible explanation is based on the general assumption that researchers recognize and strategically react to the publication bias.

It has to be stressed that the analysis presented above is rather novel and exploratory and perhaps poses more questions than it can answer. For example, I have no means to distinguish between the "crucial" and the "control" variables. Nor can we tell between manipulation checks (that tend to be highly significant) and treatment effects. Further, on some rare occasions, the null result can actually be desirable (e.g. when a control treatment is compared to past experiments to rule out the possibility that the current subject pool is highly unusual) which could result in the opposite tendencies in reporting. In this sense the effects would have probably been stronger had I been able to focus solely on “central” variables. In any case it may be a useful exercise to look for alternative explanations of the observed patterns.

The analysis may also be expanded in many interesting directions. For example, one could try to identify the "strategic reporters" to verify whether they are consistently inconsistent in different papers.

In any case, if my findings prove to be robust and putative interpretations broadly correct, they have significant implications. They seem to support the proposal to “redefine misconduct as distorted reporting” [31].

They generally call for more discipline in research, more robustness checks and more replications. As Dewald and others [32] assert "(…) errors in published articles are a commonplace rather than a rare occurrence". In the field of psychology in particular the concern for reproducibility of findings has been growing in the last years [33]. My findings also indicate that expanding study pre-registration to social sciences could be a good idea (see [34] and other papers in the Winter 2013 volume of *Political Analysis*). They further suggest the referees should look more carefully at the reported statistics, ask for raw data and inspect it carefully. Incidentally, even the revision process of this very paper involved at least two obvious mistakes on my part being found by the referees and surely many readers had similar experience.

The findings reported here may also contribute to the discussion whether we should substantially change the way we do statistics. Such calls are frequent in the literature. In his paper "The earth is round (*p*<.05)" Cohen [35] complained that "After four decades of severe criticism, the ritual of null hypothesis significance testing—mechanical dichotomous decisions around a sacred .05 criterion—still persists.". And apparently little has changed since, making the researchers call anew for abandoning the "*p*-value fallacy" [36,37]. Others suggest enforced reporting of exact *p* values instead of just the inequality [38] or forgetting about the conventional significance thresholds altogether [39]. My findings about the adverse incentive effects of the standard use of the significance thresholds indicate that these suggestions should be taken seriously.
